# Chronic diseases and multi-morbidity in persons experiencing homelessness: results from a cross-sectional study conducted at three humanitarian clinics in Germany in 2020

**DOI:** 10.1186/s12889-022-14023-w

**Published:** 2022-08-22

**Authors:** Wandini Lutchmun, Janina Gach, Christiane Borup, Guenter Froeschl

**Affiliations:** 1 Division of Infectious Diseases and Tropical Medicine, University Hospital of Munich (LMU), Munich, Germany; 2 Center for International Health, Ludwig-Maximilians-Universität, Munich, Germany; 3 Ärzte Der Welt Deutschland E.V, Munich, Germany

**Keywords:** Homelessness, Chronic diseases, COVID-19, Germany, Humanitarian aid, ETHOS classification

## Abstract

**Background:**

Persons experiencing homelessness (PEH) suffer a high burden of chronic diseases and multi-morbidity, yet face significant barriers in accessing healthcare services. These health inequalities were further aggravated during the COVID-19 pandemic. While there is a lack of comprehensive health data on PEH, even less is known about populations experiencing housing exclusion, a hidden form of homelessness. This study examines and compares chronic diseases and multi-morbidity in PEH, persons experiencing housing exclusion, and persons with secure housing who lacked access to regular healthcare services in the wake of the COVID-19 pandemic in Germany.

**Methods:**

Study participants were adults who sought medical care at clinics of the humanitarian organisation “Ärzte der Welt” in Munich, Hamburg and Berlin in 2020. The patients were categorised into three housing groups according to the ETHOS classification of homelessness and housing exclusion. Socio-demographic characteristics, self-rated health, chronic diseases and multi-morbidity were described in each group. Logistic regression analysis was used to identify socio-demographic factors associated with higher odds of chronic diseases and multi-morbidity in each housing group.

**Results:**

Of the 695 study participants, 333 experienced homelessness, 292 experienced housing exclusion and 70 had secure housing. 92.3% of all patients had either no or limited health coverage, and 96.7% were below the poverty line. Males and EU/EEA citizens were highly represented among PEH (74.2% and 56.8% respectively). PEH had lower self-rated health (47.8%, *p* = 0.04), and a higher prevalence of psychiatric illness (20.9%, *p* = 0.04). In adjusted analyses, belonging to the age group 35–49 and ≥ 50 years were associated with greater odds of chronic disease (AOR = 2.33, 95% CI = 1.68–3.24; AOR = 3.57, 95% CI = 2.55–5.01, respectively) while being ≥ 50 years old was associated with multi-morbidity (AOR = 2.01, 95% CI = 1.21, 3.33). Of the 18 participants tested for SARS-COV-2, 15 were PEH, 1 of whom tested positive.

**Conclusions:**

Housing status was not an independent risk factor for chronic disease and multi-morbidity in our study population. However, PEH reported poorer self-rated and psychiatric health. Strategies to improve access to healthcare services amongst persons experiencing homelessness and housing exclusion are needed in Germany.

## Introduction

Homelessness is a complex and dynamic state, driven by the interactions between structural factors; such as the unavailability of affordable housing, employment opportunities for low-skilled workers and income support; and individual factors, such as poverty, mental illness, substance abuse, social isolation, sexual assault and domestic violence [1, 2]. According to the German Federal Working Group on Homelessness (*Bundesarbeitsgemeinschaft Wohnungslosenhilfe*; BAG W), in 2018, 678,000 persons experienced homelessness in Germany, approximately 41,000 of whom slept without shelter, on the streets [3, 4]. Estimates show that Germany witnessed a 64.8% increase in homelessness between 2006 and 2016, mirroring the rise in most European countries in the last decade [5], and representing a growing social and public health challenge [4].

Homelessness is both a cause and a consequence of poor health [4], and persons experiencing homelessness (PEH) face mortality risks three to six times the general population [6–8]. Mental illness [9], alcohol and drug dependency [10], infectious diseases [11], and poor oral and dental health [12] are highly prevalent in this population. Recent studies have also described earlier onset and higher rates of chronic diseases and multi-morbidity in PEH compared to housed populations [13–15].

This high disease burden is a result of complex intersecting physical, mental and social burdens [16]. In addition to harsh living conditions, trauma and extreme poverty, persons experiencing homelessness must overcome significant barriers in order to receive care [16]. Lack of health insurance, organizational and bureaucratic hurdles, lack of knowledge of the healthcare system, perceived discrimination, distrust of health providers and competing priorities such as securing food and shelter, are among the many challenges PEH face in accessing, utilizing and maintaining healthcare services [16–18]. These unmet health needs result in deterioration of their health status, delayed clinical presentation and high rates of emergency department visits and hospitalisation [19, 20]. Chronic diseases are of particular concern as primary prevention measures, early detection, long-term engagement with health services and treatment compliance are often a challenge in this population [21, 22]. According to Aldridge et al., one-third of hospital deaths amongst PEH are from conditions amenable to healthcare, representing a failure of early intervention [8].

The Coronavirus Disease 2019 (COVID-19) pandemic has significantly aggravated pre-existing socio-economic and health inequalities worldwide [23]. PEH are at higher risk of infection with severe acute respiratory syndrome coronavirus 2 (SARS-COV-2) for a multitude of reasons. Homeless shelters bear high transmission risks due to crowding, shared living spaces, lack of physical distancing and high population turnover [24]. A meta-analysis of 37 studies estimated a SARS-COV-2 pooled prevalence of 32% among PEH in homeless shelters during an outbreak of COVID-19 [25]. Furthermore, a modelling study conducted by Lewer et al. described high attack rates of SARS-COV-2 in homeless shelters despite concurrent low incidence rates in the general population [26]. PEH are also less likely to access preventative measures such as regular handwashing and protective face coverings [26], and are generally less aware of and less likely to engage with public health directives, further complicated by their mobile nature [24, 27]. In addition to higher infection risks, PEH are at higher risk of severe COVID-19 disease due to their high rates of pre-existing chronic diseases and multi-morbidity [26, 28].

Due to a lack of harmonized data across Europe, assessing the extent of homelessness and identifying and addressing the needs of PEH represent a major challenge [4, 29]. In 2005, the European Typology of Homelessness and Housing Exclusion (ETHOS) was developed by the European Federation of National Organisations working with the Homeless (FEANTSA) and the European Observatory on Homelessness, to provide a common framework to define and classify homelessness [30]. While it was recommended as the official definition of homelessness in the European Union (EU) [30], ongoing inconsistencies in definition and in data collection methodologies remain [4]. Additionally, most European research on PEH is skewed towards persons sleeping rough or living in emergency shelters, excluding persons experiencing housing exclusion, such as those provisionally accommodated within institutions, with friends or family, or living in precarious housing [31]. This population referred to as the “hidden homeless”, is even more difficult to measure and likely has a different socio-demographic profile and healthcare needs [5, 32].

This study aimed to describe and compare the sociodemographic characteristics, chronic diseases, multi-morbidity levels between persons experiencing homelessness, housing exclusion and persons with secure housing, who sought care at the humanitarian clinics of “Ärzte der Welt” in 2020.

## Methods

### Study setting

The German branch of the humanitarian organisation ‘Doctors of the World’ (‘Ärzte der Welt’, ÄdW) was founded in 2000. As part of their domestic programme, they offer health services and social counselling to persons without or with limited access to the healthcare services in Germany, with the aim of (re)linking them to the regular healthcare system. Medical consultations are offered at three humanitarian clinics, located in Munich, Hamburg and Berlin. A mobile clinic in the form of a van supplied with medical amenities located in Munich, predominantly serves persons living rough or in homeless shelters. First-time presenters to the clinics are invited to a counselling session, where a trained social worker collects the patient’s socio-demographic data via questionnaires, while medical professionals collect clinical data at both initial and consecutive visits. ‘Ärzte der Welt’ publishes an annual report from the data collected, as well as more in-depth analysis of vulnerable groups or public health challenges. The annual report and analysis serve as the basis for their advocacy work and is available to political decision-makers, economic and health activists and other welfare associations. It also allows the humanitarian clinics to tailor their services to the changing needs of the population they serve.

### Study population

**Fig. 1:**
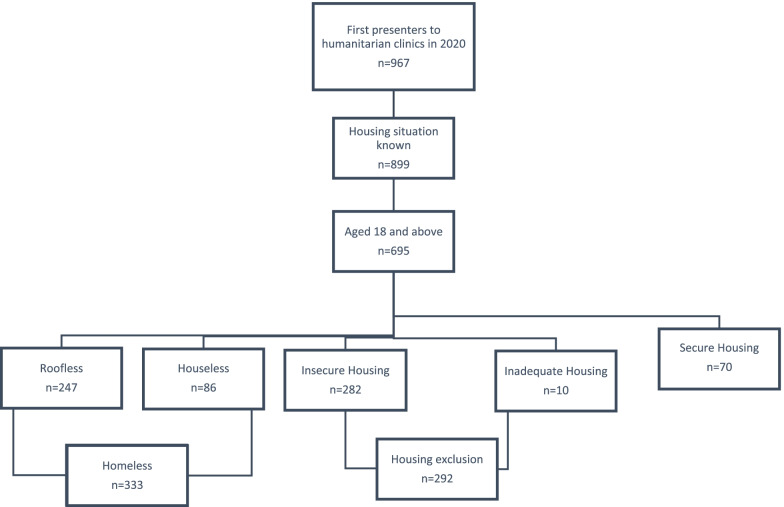
Inclusion criteria & classification of the study population as per the ETHOS classification of homelessness and housing exclusion

967 patients presented for the first time to the humanitarian clinics of ‘Ärzte der Welt’ in 2020. At presentation, 899 patients revealed their housing situation during social counselling session, of whom 695 were aged 18 and above. Our study population therefore consisted of 695 patients, who were categorised into three study groups according to the ETHOS classification of homelessness and housing exclusion: 1) Homeless 2) Housing exclusion and 3) Secure Housing. “Homeless” patients or PEH were either roofless or houseless at presentation; roofless was defined as lacking shelter of any kind and sleeping rough, and houseless as having a temporary place to sleep in a shelter or institution. “Housing exclusion” included patients with inadequate housing (living in a caravan, illegal campsites or in unfit housing) or insecure housing (without tenancy agreement, sleeping at friends’ and family or at their workplace, or living in long-term homeless accommodation). “Secure Housing” included patients with a house, apartment or room secured by a tenancy agreement. Figure 1 shows the inclusion criteria for the study populations (see Fig. 1).

### Study design and statistical analysis

We performed a retrospective, descriptive analysis of medico-administrative data collected at three humanitarian clinics and a mobile clinic of ‘Ärzte der Welt’ in Munich, Berlin and Hamburg in the year 2020. The data was entered by healthcare workers into SecuTrialR, a web-based digital data capture in clinical trials. The data was extracted and incorporated into a Microsoft Excel spreadsheet in an anonymized way. Statistical analysis was performed using Stata SE 16.

Diagnoses were inputted using the International Classification of Diseases, Tenth Revision (ICD-10); specific ICD-10 codes were created for performed SARS-COV-2 tests (U99) and SARS-COV-2 positive results (U07.1). Indication for SARS-COV-2 testing included: screening in persons living in shared accommodation, including homeless shelters, (denoted as ICD-10 code Z11) or in persons exhibiting respiratory symptoms suggestive of COVID-19 infection (denoted as J06.9, J12.8 or R43, among others). The variable “chronic diseases” was defined as any ICD-10 diagnosis matched with the variable “chronic”, which was inputted by the medical professional at each medical consultation. The variable “Psychiatric illness” was created by matching all the ICD-10 codes within the group “F” (Mental, Behavioural and Neurodevelopmental Disorders) with the variable “Chronic”. Multi-morbidity was defined as the occurrence of 2 or more chronic diseases. Age was categorised into 3 groups: 1) 18–34 years 2) 35–49 years and 3) > 50 years.

Frequencies were used for categorical variables and medians with inter-quartile range for the non-normally distributed continuous variables. The correlation between categorical variables was done by the Chi-square test or Fisher’s exact test for small numbers. Logistic regression was performed to identify socio-demographic factors associated with higher risk of chronic disease and multi-morbidity. The multiple logistic regression model included all socio-demographic factors potentially contributing to the prevalence of chronic illness and multi-morbidity in the study population. Patients who refused to answer questions, had missing data or responded “I don’t know” to questions were not included in the analyses. The threshold of statistical significance was set at an alpha level of 0.05.

## Results

I. **Sociodemographic characteristics**

In 2020, 695 patients aged 18 and above who presented to a humanitarian clinic gave information about their housing situation. 333 (47.9%) patients were categorised as experiencing homelessness, 292 (42.0%) as experiencing housing exclusion and only 70 patients (10.1%) had secure housing. Table 1 summarises the socio-demographic characteristics of the population groups (Table 1). Significant differences were noted between the patient groups in all socio-demographic characteristics (Table 1). Among patients experiencing homelessness, the majority were male (74.2% vs 25.8% females), between 35–49 years old (41.4%) and European Union/European Economic Zone (EU/EEA) nationals (56.8%). The majority of PEH originated from Romania (18.2%), followed by Bulgaria (15.2%), Poland (7.6%), and Hungary (4.9%). Females and non-EU/EEA nationals made up a higher proportion of patients experiencing housing exclusion compared to the other groups (54.6% and 60.5%, respectively). Patients originating from Serbia (15.2%), Bulgaria (14.2%), Vietnam (8.3%), Romania (5.2%) and Albania (3.81%) were highly represented in this group. In all three groups, the large majority of patients lived below the poverty line; defined in 2020 in Germany as earning less than 1136 euros per month; and were entitled to either no or partial health coverage.

**Table 1:** Socio-demographic characteristics of patients experiencing homelessness, housing exclusion and with secure housing in Germany in 2020

Characteristics	Homeless N (%)	Housing exclusion N (%)	Secure Housing N (%)	*P* value*
**Gender**				
Male	247 (74.17)	132 (45.36)	37 (53.62)	< .001
Female	86 (25.83)	159 (54.64)	32 (45.38)	
**Age category**				
18–34	116 (34.83)	116(39.73)	33(47.14)	0.01
35–49	138 (41.44)	88 (30.14)	18 (25.71)	
≥50	79 (23.72)	88 (30.14)	19 (27.14)	
**Country of origin**				
German National	33 (10.03)	30 (10.38)	18 (25.71)	< .001
EU/EEA National	187 (56.84)	84 (29.07)	21 (30.00)	
Non-EU/EEA National	109 (33.13)	175 (60.55)	31 (44.29)	
**Immigration status**				
Undocumented migrant	17 (5.33)	111 (39.64)	6 (8.70)	< .001
Other	302 (94.67)	169 (60.36)	63 (91.30)	
**Income bracket**				
Below poverty line	304 (97.12)	273 (98.20)	60 (88.24)	< .001
Above poverty line	9 (2.88)	5 (1.80)	8 (11.76)	
**Insurance status**				
No Health Coverage	221 (66.97)	248 (87.94)	46 (67.65)	< .001
Partial Health Coverage	65 (19.70)	31 (10.99)	17 (25.00)	
Full Health coverage	44 (13.33)	3(1.06)	5(7.35)	

^*^p-values were determined by the chi-square test for independence

II.  **Self-rated health**

**Fig. 2:**
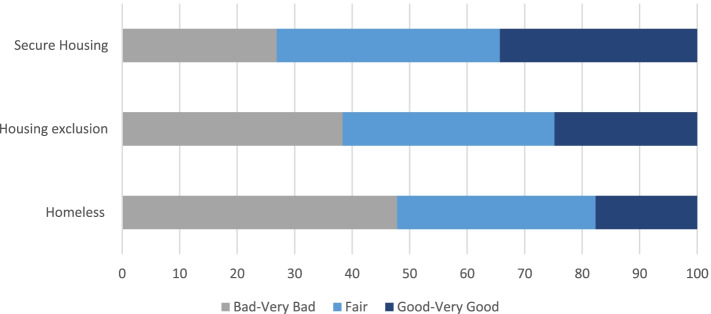
Self-rated health among patients experiencing homelessness (*n* = 113), housing exclusion (*n* = 266) and with secure housing (*n* = 67) at first presentation to a humanitarian clinic in Germany in 2020

When asked to rate their general state of health, a higher proportion of patients experiencing homelessness (54/113; 47.8%) rated their health as “bad” or “very bad” in comparison to patients experiencing housing exclusion and patients with secure housing (38.4% and 26.8% respectively). Only 17.7% (20/113) of PEH described their general health status as “good” or “very good” (vs 24.8% and 34.3%; *p* = 0.04; see Fig. 2).

III. **Chronic Diseases**

Chronic diseases made up 45.7% (459/1,004) of the total number of medical diagnoses made among the 695 adult patients attending the humanitarian clinics in 2020. 43.3% (220/508) of diagnoses in PEH were chronic, compared to 47.8% (190/397) in patients experiencing housing exclusion and 49.5% (49/99) in patients with secure housing (*p* = 0.29). Table 2 ranks the three most common chronic diagnoses in each population (see Table 2). Hypertension was the most diagnosed chronic disease in all three groups, with no significant association with a particular housing situation (*p* = 0.34). This was followed by Diabetes Mellitus amongst persons experiencing housing exclusion (8.42%) and with secure housing (6.12%), and by ‘Reaction to severe stress and adjustment disorders’ in PEH (5%), which included the main diagnosis of Post-Traumatic Stress Disorder (PTSD). Alcohol-related disorders were only diagnosed amongst PEH (9/220; 4.09%, *p* = 0.10).

**Table 2:** The three most common chronic diagnoses among persons experiencing homelessness, housing exclusion and persons with secure housing, n (%)

Homeless (*n* = 220) (N; %)	Housing Exclusion (*n* = 190) (N; %)	Secure Housing (*n* = 49) (N; %)
Hypertension (22; 10.00%)	Hypertension (27; 14.21%)	Hypertension (4; 8.16%)
Reaction to severe stress and adjustment disorders (11; 5.00%)	Diabetes Mellitus Type 2 (16; 8.42%)	Diabetes Mellitus Type 2 (3; 6.12%)
Alcohol-related disorders (9; 4.09%)	Chronic Ischemic Heart Disease (6; 3.16%)	Depression (3; 6.12%)

Table 3 describes the frequency of five common chronic diseases among the three housing groups. For the purpose of comparison, the variable “Psychiatric illness” was created by combining all chronic diagnoses within the ICD-10 group “F” (Mental, Behavioural and Neurodevelopmental disorders). Chronic Obstructive Pulmonary Disease (COPD) and Asthma were combined into one variable. There was a significant association between chronic psychiatric illness and homelessness (*p* = 0.04).

**Table 3:** Frequency of five common chronic diseases among persons experiencing homelessness housing exclusion and persons with secure housing, N (%)

Chronic disease	Homeless (*n* = 220) N (%)	Housing Exclusion (*n* = 190) N (%)	Secure Housing (*n* = 49) N (%)	*P*-value*
Hypertension	22 (10.00%)	27 (14.21%)	4 (8.16%)	0.34
Diabetes Mellitus Type 2	8 (3.64%)	16 (8.42%)	3 (6.12%)	0.10
Chronic Ischemic Heart Disease	4 (1.82%)	6 (3.16%)	2 (4.08%)	0.48
COPD/Asthma	13 (5.91%)	5 (2.63%)	3 (6.12%)	0.20
Psychiatric illness	46 (20.91%)	24 (12.63%)	12 (24.49%)	0.04

^*^p-values determined by Fischer’s exact test

Table 4 summarises the results of the single covariate and multiple variable logistic regression analysis identifying socio-demographic factors associated with the prevalence of a chronic disease. Factors significantly associated with increase odds of chronic disease in bivariate analysis; adjusted for age as a confounder; were being a non-EU/EEA national (OR 1.52, CI 95% = 1.02, 2.26) and belonging to the age groups 35–49 years (OR 1.95, 95% CI = 1.43, 2.64) and ≥50 years (OR 3.29, 95% CI = 2.41, 4.47). Age was the only factor that increased odds of a chronic disease in the multiple covariates adjusted models (AOR 2.33, 95% CI = 1.68, 3.24 & AOR 3.57, 95% CI = 2.55, 5.01 in age groups 35–49 and ≥50 respectively).

**Table 4:** Factors associated with chronic disease amongst persons experiencing homelessness, housing exclusion and persons with secure housing in Germany in 2020

	Single covariate analysis		Multiple covariates adjusted analysis	
	OR (95% CI)	*p*-value	OR (95% CI)	*p*-value
**Housing situation**^a^				
Homeless (Ref)	1.00		1.00	
Housing exclusion	1.14 (0.87, 1.50)	0.172	1.08(0.79, 1.49)	0.611
Secure housing	1.35 (0.87, 2.12)	0.258	1.51(0.94, 2.44)	0.089
**Age group**				
18–34 (Ref)	1.00		1.00	
35–49	1.95 (1.43, 2.64)	< .001	2.33(1.68, 3.24)	< .001
≥50	3.29 (2.41, 4.47)	< .001	3.57(2.55, 5.01)	< .001
**Gender**^a^				
Female (Ref)	1.00		1.00	
Male	0.93 (0.72, 1.19)	0.553	1.01 (0 .76, 1.34)	0.956
**Country of origin**^a^				
German National (Ref)	1.00		1.00	
EU/EEA National	1.16 (0.79, 1.71)	0.438	1.11(0.71, 1.73)	0.646
Non-EU/EEA National	1.52 (1.02, 2.26)	0.039	1.45 (0.94, 2.24)	0.094
**Insurance**^a^				
Full Health coverage (Ref)	1.00		1.00	
Partial Health Coverage	0.96(0.56, 1.65)	0.890	0.94 (0.54, 1.62)	0.818
No Health Coverage	0.90 (0.57, 1.43)	0.657	0.89(0.55, 1.46)	0.665

^a^all factors in single covariate analysis were adjusted for age as a possible confounder

IV. **Multi-morbidity**

Multi-morbidity was found in 44.96% (58/129) of PEH, 40.9% (45/110) of patients experiencing housing exclusion and 44.44% (12/27) of patients with secure housing (*p* = 0.813). Belonging to the age group ≥50 years old was the only factor associated with a higher likelihood of multi-morbidity (OR 2.01, 95% CI = 1.21, 3.33) in single covariate analysis (see Table 5).

**Table 5:** Factors associated with multi-morbidity in persons experiencing homelessness, housing exclusion and persons with secure housing in Germany in 2020

	Single covariate analysis	
	OR (95% CI)	*p*-value
**Housing situation**		
Homeless (Ref)	1.00	
Housing exclusion	0.92 (0.61, 1.38)	0.68
Secure housing	1.08 (0.55, 2.11)	0.82
**Age group**		
18–34 (Ref)	1.00	
35–49	0.78 (0.48, 1.28)	0.33
≥50	2.01 (1.21, 3.33)	0.01
**Gender**		
Female (Ref)	1.00	
Male	0.70 (0.47, 1.05)	0.09
**Country of origin**		
German National (Ref)	1.00	
EU/EEA National	1.11 (0.59, 2.08)	0.37
Non-EU/EEA National	0.84 (0.45, 1.57)	
**Insurance**		
Full Health coverage (Ref)	1.00	
Partial Health Coverage	1.26 (0.54, 2.94)	0.58
No Health Coverage	0.99 (0.48, 2.01)	0.97

V. **SARS-COV-2**

The humanitarian clinics began testing for SARS-COV-2 infection using PCR (polymerase chain reaction) tests from April 2020 and rapid antigen tests from October 2020. Data regarding SARS-COV-2 testing was only available from the Munich clinics. Only 18 patients received SARS-COV-2 testing, of whom 1 tested positive. 5 (27.7%) patients were tested as part of screening measures; 4 of whom were PEH; the rest were symptomatic. There were no SARS-COV-2 outbreaks reported in any of the clinics in 2020.

## Discussion

This study compares chronic diseases and multi-morbidity between patients of different housing circumstances, who sought medical care at clinics of a humanitarian network in the wake of the COVID-19 pandemic. Our findings showed that homelessness and housing exclusion were not associated with a higher likelihood of chronic diseases and multi-morbidity. However, we report poorer self-rated health and a higher prevalence of psychiatric illnesses amongst PEH, as well as noteworthy socio-demographic differences between the three housing groups.

The majority of patients who sought care at the humanitarian clinics had either no or limited health coverage (92.3%) and were below the poverty line (96.7%). While selection bias contributes to this finding, it highlights the role of health insurance and social exclusion as important barriers to accessing regular healthcare services in Germany. Interestingly, a higher proportion of PEH had health coverage (33.03%) compared to persons experiencing housing exclusion (12.1%). A possible explanation is that a larger proportion of PEH in our study originated from EU/EEA member states and may have been eligible for insurance entitlements from their respective countries. Despite their entitlements, they preferred the services of a humanitarian organisation, which may reflect the multitude of individual barriers faced by this population, but also the benefit of the trauma-informed care and social counselling services provided by the clinics.

About three times as many men (74.2%) in our study experienced homelessness in comparison to women. The predominance of men among PEH has been widely reported [14, 19, 33, 34]. However, more than half (54.6%) of persons experiencing housing exclusion were women. This is consistent with previous reports that women are more likely to rely on relatives, friends, and other informal systems when they fall into homelessness, only approaching homeless and welfare services when these supports are exhausted [35]. Women are shown to be present in larger proportions when definitions of, and data collection frameworks on homelessness extend beyond persons living in emergency shelters and sleeping rough [5]. Homelessness among women is also commonly triggered by experiences of intimate partner violence (IPV) [35], which underwent a dramatic increase as a result of the lockdowns and restrictions imposed during the COVID-19 pandemic [36, 37]. A higher proportion of PEH also originated from EU/EEA countries (56.8%), while non-EU/EEA nationals (60.5%) and undocumented migrants (39.6%) were more highly represented in persons facing housing exclusion. According to previous reports by the BAG W, EU/EEA citizens make up most rough sleepers in Germany, consistent with our findings [3]. The overall high migrant population in our study population also highlights the difficulties faced by individuals with a migrant background in accessing regular health services in Germany. Moreover, it confirms a previous statement by the European Observatory on Homelessness describing migration as a new structural risk factor for the development of homelessness [38].

Chronic diseases made up 45.7% of the total number of diagnoses in patients visiting the humanitarian clinics in 2020. The predominance of hypertension, diabetes and chronic ischemic heart disease in our study population mirrors the most common chronic diseases in the general German population [39]. However, the prevalence of hypertension (10%, 14.2% and 8.2% in PEH, persons experiencing housing exclusion and living in secure housing, respectively) was considerably lower than the 12-month prevalence of 31.8% in the general population in Germany, reported by the German Health Update (GEDA), a population-representative health survey conducted by the Robert Koch Institute (RKI) [40]. Similarly, the prevalence of chronic ischemic heart disease is higher in the general population; 5.8% compared to 1.82%, 3.16% and 4.1% in PEH, persons experiencing housing exclusion and with secure housing, respectively [39]. The 12-month prevalence of diabetes (8.9%) was comparable to our population experiencing housing exclusion (8.4%), but higher than the two other housing groups [39]. These findings could be attributed to the majority of the diagnoses being new rather than previously diagnosed and self-reported by the patients. Furthermore, since chronic diseases develop over time and the manifestations are often intermittent, a “point” prevalence based on a single examination and clinic visit is likely to underestimate disease frequency.

Our analysis found that age was the only factor associated with a higher risk of chronic disease, reflecting the epidemiological nature of most chronic diseases. In contrast to our findings, multiple cohort studies have demonstrated higher rates of chronic diseases and multi-morbidity in persons experiencing homelessness [14, 41–43]. A study analysing electronic health records (EHRs) in the UK between 1998 and 2019 found that persons experiencing homelessness were 1.8 times more likely to have baseline prevalence of cardiovascular diseases (CVD), and suffered a higher burden of comorbidities in comparison to housed controls [15]. A systematic review of 17 observational studies on CVD in homeless versus housed individuals also found that hypertension was more likely to occur in persons experiencing homelessness [21]. However much of the research draws comparisons between PEH and the general or generally deprived population, without accounting for the additional barriers faced by these populations. A study examining the unmet health needs in homeless versus vulnerably housed adults in three Canadian cities, reported no significant differences between the two, suggesting they are intersecting populations with similar health status and experienced barriers [17].

However, we describe a positive association between psychiatric illness and homelessness, consistent with numerous studies describing higher rates major depression, anxiety disorders, bipolar disorder and alcohol and drug dependency among PEH [1, 4, 9, 18, 44]. While the association between alcohol-related disorders and housing situation was not significant, only PEH in our study were diagnosed with the former. Two systematic reviews spanning studies published in 1979–2005 and 2007–2021, and altogether comprising 13,733 individuals, described substance-use disorders, in particular alcohol-related disorders as the most common psychiatric illness among PEH, with random effects pooled prevalence of 37.9% and 36.7%, respectively [44, 45]. Interestingly, the latter study reported studies conducted in Germany were associated with higher prevalence rates in multivariable analysis [45]. Similarly, a systematic review of studies conducted in Germany reported a pooled prevalence of alcohol dependency of 55.4%; higher than any other psychiatric illness, and 22 times higher than the prevalence in the general German population [46]. The low prevalence of alcohol-dependency in our study (4%) may result from multiple factors. Our study population included first presenters to the clinics, and potentially excluded a larger population who visited the psychiatric outpatient services of the clinic, or were linked into the regular mental health services after their first visit. Furthermore, clinicians at the humanitarian clinics may not have been specifically trained at diagnosing mental illness, and relied on clinical examination only to reach a diagnosis, which could have resulted in potential underdiagnoses.

Psychiatric illnesses are an important contributor to the increased mortality rates among PEH, from suicide and substance abuse, but also through higher rates of criminalisation and violent victimisation [44]. Psychiatric illnesses also further complicate the treatment of chronic diseases by acting as an important barrier to seeking and maintaining contact with health services [22].

### Strengths and limitations

There are several strengths and limitations to this study. Through our cooperation with the clinics of “Ärzte der Welt”, we were able to study a population which is generally hard to reach and often excluded from operational and medical research. Our study also includes persons experiencing housing exclusion, a population often excluded in studies examining homelessness. While our findings cannot be generalised to the entire population experiencing social exclusion and homelessness in Germany, we provide insight into a population deprived of access to regular health services at one point in time. We acknowledge that the characteristics of our study population are likely influenced by public health measures implemented within the period of our study, and due to the lockdowns and restrictions imposed throughout 2020, it is likely that it excludes an even larger population experiencing marginalisation who might have even higher mental and physical health needs. Furthermore, some humanitarian clinics underwent restructuring, temporary closure, or experienced understaffing, affecting data collection and resulting in underreporting. Systematic data collection regarding referrals and testing were also not fully implemented in the clinics until late 2020, with some clinics not undertaking SARS-COV-2 testing at all, contributing to the scarcity of data on SARS-COV-2. However, this underlines an important effect of the COVID-19 pandemic on service provision for persons experiencing social exclusion.

Another limitation is the much smaller population of patients with secure housing in comparison to the other groups, affecting the power of our statistical analysis. This is an expected finding, as persons visiting the humanitarian clinics are more likely to originate from low socio-economic backgrounds, and therefore experience precarious housing. Due to a hesitancy to disclose sensitive information, some patients also did not provide answers to all questions, resulting in missing data. Additionally, since we relied on the physician’s coding of a disease as “acute” or “chronic” during medical consultations, some conditions may not have been coded or coded inappropriately, resulting in underdiagnoses. Physicians work at the humanitarian clinics on a voluntary and rotational basis and may not have the necessary training to ask for specific mental health experiences, such as exposure to domestic violence, adverse childhood experiences and addiction disorders, which fit within ICD-10 diagnoses but were not identified in our population.

## Conclusion

Our study provides a snapshot into the chronic health of persons who required the medical services of a humanitarian organisation in 2020. While housing situation was not an independent risk factor for chronic diseases in this study, we highlight the need for a shift in focus of research to chronic diseases, which contribute to a high total burden of disease and premature mortality in the ageing homeless population [21].

Humanitarian organizations often lack the resources to collect systematic and in-depth data on the populations they serve. Furthermore, since they aim to link patients to the formal healthcare system, follow-up data on the progression of chronic diseases, complications, emergency visits and hospital stays are unavailable in these populations. This emphasizes the need for healthcare workers at all levels of care to take a thorough social history, which would increase the visibility of persons experiencing social exclusion and likely reveal a wider range of physical, mental and social health conditions.

The lack of an official definition and nationwide statistics on homelessness in Germany [5] further contributes to the challenges in assessing the needs of persons experiencing homelessness. Moreover, due to the absence of a national strategy on homelessness, significant regional disparities in service provision for PEH exist, which was amplified in 2020. Our analysis suggests including persons experiencing housing exclusion in existing definitions of homelessness, as they encompass a form of homelessness with similar needs and barriers to healthcare. Furthermore, widening the definition beyond persons sleeping rough and in shelters is likely to reveal more women and a different migrant profile, who would benefit from adapted supports [32].

While our study did not attempt to propose interventions to improve the chronic health of the homeless, we suggest that addressing the underlying social exclusion is key in improving the health of persons experiencing homeless. The intersections of homelessness, chronic diseases and the COVID-19 pandemic represent an emerging public health crisis in high-income countries.

## Data Availability

The data generated and analysed during this study are available from the corresponding author on reasonable request.

## Abbreviations

ÄdW: Ärzte der Welt
BAG W: The Federal Working Group on Homelessness
CVD: Cardiovascular disease
COVID-19: Coronavirus disease 2019
EHR: Electronic health record
ETHOS: European Typology of Homelessness and Housing Exclusion
EU: European Union
EU/EEA: European Union/European Economic Zone
FEANTSA: European Federation of National Organisations working with the Homeless
GEDA: German Health Update
ICD-10: International Classification of Diseases, Tenth Revision
IPV: Intimate Partner Violence
NGO: Non-governmental organisation
RKI: Robert Koch Institute
SARS-COV-2: Severe Acute Respiratory Syndrome Coronavirus 2
SRH: Self-rated Health
PCR: Polymerase Chain Reaction
PEH: Persons Experiencing Homelessness
PTSD: Post-Traumatic Stress Disorder
US: United States
